# Antibiotic prescription patterns for treating dental infections in children among general and pediatric dentists in teaching institutions of Karachi, Pakistan

**DOI:** 10.1371/journal.pone.0235671

**Published:** 2020-07-10

**Authors:** Sadaf Ahsan, Muhammad Zafar Iqbal Hydrie, Syed Muhammad Zulfiqar Hyder Naqvi, Munir Ahmed Shaikh, Muhammad Zahid Shah, Syed Imtiaz Ahmed Jafry

**Affiliations:** 1 Baqai Dental College, Baqai Medical University, Karachi, Pakistan; 2 Department of Community Medicine, Baqai Medical University, Karachi, Pakistan; 3 Department of Family Medicine, Baqai Medical University, Karachi, Pakistan; Federal University of Sergipe, BRAZIL

## Abstract

**Background:**

Antibiotics are regularly prescribed by dental professionals in their practice, for the purpose of dental treatment as well as for the prevention of infection. The inappropriate use of antibiotics is a significant factor in the rise of antibiotic resistance. There is an immediate need for the advancement of prescribing guidelines and instructive polices to encourage the rational and appropriate utilization of medications especially antibiotics in dentistry.

**Objective:**

The aim of this study was to identify the frequency of antibiotic prescription for treating dental infections in children among dentists in teaching institutions of Karachi, Pakistan and whether they are adhering to the prescribed international guidelines.

**Methods:**

A cross-sectional study was conducted in three private and two public colleges of Karachi. After taking written informed consent and checking the inclusion criteria, a total of 380 participants were interviewed using a pre-designed validated questionnaire which included demographic profile and clinical case scenarios. Data were entered and analyzed on SPSS version 20. Inferential analysis was performed using chi-square test. The significance level was set at 0.05.

**Results:**

Of the 380 subjects, a majority (71.3%) treated 15 or less children per month (n = 271) while 28.7% of dentists (n = 109) treated more than 15 children per month. Overall adherence to American Academy of Pediatric Dentistry guidelines was low from 26.1% to 44.2%. The difference between adherence of dentists with low and high volume of pediatric patients was significantly different for case scenarios 1, 3, 4 and 5 (p<0.001 for all) where dentists who treated 15 or less children per month were more likely to be adherent to standard antibiotic prescription guidelines than those who treated more than 15 children per month.

**Conclusions:**

This study shows that majority of dentists, particularly dentists with high volume of pediatric patients lacked adherence to professional guidelines for prescribing antibiotics for treating dental infection in children. There seem to be a lack of harmony between the recommended professional guidelines and the antibiotic prescribing pattern of dentists. Regular updates and continuing medical education for the health professionals regarding comprehensible and specific professional guidelines may lead to improved adherence of antibiotics prescription amongst dentists.

## Introduction

During the last few decades one of the most common drugs prescribed in both children and adults has been antibiotics as they significantly reduce human mortality and morbidity [1]. From 2000 to 2015, the global antibiotic consumption in terms of defined daily doses has seen an increase of 65%, mainly in low middle income countries, such as Pakistan [2]. Thus use of antibiotics in developing countries has been observed to be much higher compared to the developed world [3–5]. One probable cause could be extended as well as unnecessary use of broad spectrum antibiotics in these countries [6,7]. Prescription of broad-spectrum antibiotics to attain the patient’s satisfaction even if antibiotics are not indicated is also common in these places [8]. Inappropriate prescription of antibiotics leads to adverse drug reaction and emergence of drug resistant organisms, stretching the already limited health care resources in developing countries [9, 10]. This leads to human sufferings along with substantial added economic burden [11]. Some health care professionals only concentrate on treating the present symptoms without concern about future antibiotics resistance aggravating the problem [12, 13].

The growing threat of antibiotic resistance is linked to a wide range of factors and does not simply indicate that the body is becoming vulnerable to infections, rather micro-organisms are becoming resistant to the antibiotics used to eliminate them [14]. Antibiotic resistance takes place when microorganisms adapt and evolve as "superbugs" against antibiotics, making previous medications ineffective to kill them and continue to survive in the body becoming a danger of spread to other people [15]. Inadequate and extravagant utilization of antibiotics is one of the significant factors in the rise of antibiotic resistance [16]. The increasing emergence of antibiotic-resistant bacteria is becoming one of the major threats to public health in the 21^st^ century and with this global rise there is need for appropriate and proper use of antibiotics [17]. Antibiotics resistance has been recognized as a major issue in health care and improper prescription of anti-microbial substances (antibiotics) by experts is a major concern in medical as well as dental specialties [18]. The awareness about the threat of antibiotic resistance among healthcare workers, particularly dentists who frequently prescribe antibiotics for treatment and prevention of various dental infections in not uniform. Antibiotics are commonly used in dental practice due to anticipation of contaminating dental procedures because of infections and a majority of dental prescriptions in UK happen to be for antibacterial drugs [19].

The literature provides evidence of inadequate practices by dentists, manifested by over-prescribing antibiotics due to inadequate knowledge or social factors [20]. Another survey done in UK reported that around 15% of dentists recommended antibiotic use on daily basis while 40% of the dentists prescribed antibiotics somewhere around three cases per week [21]. Another cross-sectional survey conducted in Saudi Arabia amongst dentists on prescription patterns of antibiotics showed extensive variety of recommending antibiotics among dentists [22]. Similarly, in Istanbul-Turkey a study showed conflicting antibiotic prescription with unnecessary anti-microbial utilization and inadequate drug usage in patients without following any professional guidelines [23]. A survey done in America on assessing antibiotic use for treating dental infections in children, showed only a quarter of dental professionals and pediatric dentists adhered to the professional American Academy of Pediatric Dentistry (AAPD) guidelines for endorsing antibiotics [24]. A propensity for overuse of antibiotics, without clinical indication, has thus been reported among members of the American Academy of Pediatric Dentistry [25].

Similar trends are also observed in developing countries, where a substantial proportion of dentists prescribe antibiotics for non-indicated clinical conditions [26]. Regionally an indian survey on the use of antibiotics showed that there was over antibiotics endorsing in the absence of following any expert rules, which could be a precedent leading to the global issue of antibiotic resistance [27]. Most dental infections in children are bacterial in origin but only a limited number require antibiotics since most of these infections respond very well to operative procedures such as removing the source of infection and when antibiotics are needed, they are used as an adjunct to the operative therapy instead of been used as the only line of treatment [28]. It was observed in a study done in Saudi Arabia that while recommending antibiotics for odontogenic infections in children, there was an absence of constant adherence to the professional guidelines amongst the dentists [24]. Abuse in prescription of antibiotics, such as inappropriate dosing regimens and longer than needed periods of prescription might contribute to the emergence of antibiotic resistance among children. As a result oral cavities of very young children have also been reported to have multidrug resistant bacteria [29, 30]. Unfortunately, there is scarcity of resources on dental antibiotic prescribing guidelines in general and for children particularly.

In this study we hoped to measure the frequency of antibiotics prescription for treating dental infections in children among dentists in teaching institutions of Karachi, Pakistan. We also aimed to assess if the dental health professionals were following the recommended professional guidelines such as the AAPD guidelines, so as to develop more improved and unambiguous antibiotic prescription guidelines especially for children if needed.

## Materials and methods

A cross-sectional study was carried out at five public and private tertiary care dental teaching institutions of Karachi from November 2018 to May 2019 namely Baqai Dental College, Altamash Institute of Dental Medicine, Bahria University and Dental College, Abbasi Shaheed Hospital and Dow International Medical College. Ethical clearance was obtained from the parent institution with reference number No. FHM30a-2019/MPH student/ Batch26. Using the percentage frequency of antibiotic prescription among dentists from a previous study i.e. 44.2%, with 95% confidence level and 5% precision, the minimum required sample size was calculated to be 380 participants using the formula ‘n = z^2^*(p)(1-p)/c^2’^ where z = 1.96 for 95% CI, p = 0.442 and c = 0.05. Dentists who were registered with the National Dental Council and gave informed consent were included in the study whereas those who refused to give written informed consent were excluded from the study. After checking eligibility, the dentists were approached using convenient sampling technique for inclusion in the study.

Dentists were considered as having high volume of pediatric patients if they used to see more than 15 children per month or as having low volume of pediatric patients if they saw 15 or less children per month. The participants were asked to fill a pre-tested and validated questionnaire designed to record socio-demographic details and information related to their antibiotics prescribing pattern. The questionnaire was taken from an earlier study done by Cherry WR et al in 2012 [24]. The questionnaire consisted of two main sections; the first section consisted of demographic variables while the second section consisted of five different clinical case scenarios developed by the American Academy of Pediatric Dentistry (AAPD) to explore the rationality of antibiotics use [24]. These clinical case scenarios are as follows: Case 1—A healthy (ASA I) 9-year-old child, who is a patient of record, visits your office during regular business hours with tooth pain in the lower right quadrant. On clinical examination, you notice a deep carious lesion on tooth number 85 (mandibular right primary second molar); Case 2—A healthy (ASA I) 9-year-old child, who is a patient of record, visits your office during regular business hours with tooth pain in the lower right quadrant and a fever of 101 F. On clinical examination, you notice a deep carious lesion on tooth number 85 (mandibular right primary second molar); Case 3—A healthy (ASA I) 9-year-old child, who is a patient of record, visits your office during regular business hours with tooth pain in the lower right quadrant. The child has no fever. On clinical examination, you notice a deep carious lesion on tooth number 85 (mandibular right primary second molar) along with a draining fistula; Case 4—The parent of a healthy (ASA I) 9-year-old child, who is a patient of record, calls you on a Saturday afternoon because the child has a chief complaint of tooth pain in the lower right quadrant; and Case 5—The parent of a healthy (ASA I) 9-year-old child, who is a patient of record, calls you on a Saturday afternoon and reports that the child has pain on the lower right quadrant with some warmness of the skin and some swelling that she noticed that morning. The adherence of the participating dentists to AADP guidelines was assessed by means of interview and evaluating their responses regarding antibiotic prescription in each of the five different clinical case scenarios described for being in line with the AADP guidelines.

Data were entered and analyzed in statistical package for social science (SPSS) version 20 whereas graphs and tables were made by MS (Microsoft) Excel. Descriptive analysis such as frequencies and percentages were generated for categorical variables while means and standard deviation were calculated for continuous variable. Inferential analysis was performed using chi square test to explore associations of studied independent variables with the study outcome. The significance level was set at 0.05.

## Results

A total of 380 participants were included in the study with a response rate of 100%. Table 1 shows the demographic and practice characteristics of the ting dentists in the study. The mean age of the participants was 25.12 ± 3.4 years while 69.7% (n = 265) of them were females. Majority (71.3%) of them treated 15 or less children per month (n = 271) while 28.7% of them (n = 109) treated more than 15 children per month. Moreover, 63.9% (n = 342) of the dentists were seeing patients in outpatient department in public sector and 55.0% (n = 209) were working in hospital dentistry. The table also shows the general pattern of antibiotics prescription with 19.7% (n = 75) of the dentists prescribing antibiotics daily while 48.9% (n = 185) of them were prescribing it weekly. The most commonly prescribed antibiotic in this study was amoxicillin (n = 349, 91.8%).

**Table 1:** Demographic and practice characteristics of study sample (n = 380).

Variables (n = 380)	Frequency(%)
**Age (Years)**	25.12±3.4
**Age Group**	
<25 Years	230(60.5)
25 Years or Above	150(39.5)
**Gender**	
Male	115(30.3)
Female	265(69.7)
**Educational qualification**	
General dentists	338(88.9)
Pediatric dentists	42(11.1)
**Child treated per month**	
≤15	271(71.3)
>15	109(28.7)
**Public practice**	
No	137(36.1)
Yes	243(63.9)
**Academic practice**	
No	166(43.7)
Yes	214(56.3)
**Hospital dentistry**	
No	171(45.0)
Yes	209(55.0)
**Primary health centers**	
No	365(96.1)
Yes	15(3.9)
**How often prescribe antibiotic**	
Daily	75(19.7)
Weekly	185(48.9)
Monthly	88(23.2)
Hardly ever	31(8.2)
**Antibiotic type amoxicillin**	
No	31(8.2)
Yes	349(91.8)
**Antibiotic type penicillin**	
No	211(55.5)
Yes	169(44.5)
**Antibiotic type clindamycin**	
No	317(83.4)
Yes	63(16.6)
**Antibiotic type cephalexin**	
No	349(91.8)
Yes	31(8.2)

Table 2 presents dentists response to the five clinical case scenarios as per AAPD professional guidelines. Case 1 represents the collective symptoms of facial swelling, pain and radiographic evidence of pathology. Overall, 26.8% of dentists in the study were in adherence with the professional guidelines. When fever was added to the list of symptoms as in Case 2, the overall adherence was similar at 26.1%. When local swelling was added and fever removed from symptoms as in Case 3, overall adherence to the professional guidelines increased to 36.8% in the study. Case 4 and Case 5 were considered as weekend cases with overall 44.2% of the dentists in case 4 adherent with the professional guidelines where you would see the patient before prescribing antibiotics and prescribe antibiotics only for pain and facial swelling. While in Case 5 adherence with the professional guidelines decreased to 33.9% for the dentists in the study when warmness of skin was included. Moreover, it was also seen that the difference between adherence of dentists with low and high volume of pediatric patients was significantly different for case scenarios 1, 3, 4 and 5 (p<0.001 for all) where dentists who treated up to 15 children per month were more likely to be adherent to standard antibiotic prescription guidelines than those who treated more than 15 children per month.

**Table 2:** Responses to clinical scenarios: Adherence to professional guidelines.

Clinical Scenarios and Correct Responses	Overall Adherence to Guidelines (n = 380)	Dentists with low volume of pediatric patients (≤ 15 children treated per month) (n = 271)	Dentists with high volume of pediatric patients (> 15 children treated per month) (n = 109)	p
	Frequency (%)	Frequency (%)	Frequency (%)	
**Case 1** Prescribe antibiotics only for pain, facial swelling and radiographic evidence of pathology	102(26.8)	88(32.4)	14(12.8)	<0.001
**Case 2** Prescribe antibiotics only for pain, facial swelling and radiographic evidence of pathology	99(26.1)	77(28.4)	22(20.2)	0.098
**Case 3** Prescribe antibiotics only for pain, facial swelling and radiographic evidence of pathology	140(36.8)	115(42.4)	25(22.9)	<0.001
**Case 4** Would see patient before prescribing antibiotics and prescribe antibiotics only for pain and facial swelling	168(44.2)	148(54.6)	20(18.3)	<0.001
**Case 5** Would see patient before prescribing antibiotics and prescribe antibiotics only for pain and facial swelling	129(33.9)	117(43.1)	12(11.0)	<0.001

## Discussion

This study investigated the trends in antibiotic prescription pattern and the awareness of antibiotic prescribing guidelines among dentists in various dental colleges of Karachi, Pakistan and assessed their adherence to the AAPD professional guidelines for children.

We found that over seventy percent dentists treated up to 15 children per month and as such were considered as dentists with low volume of pediatric patients as compared to those who treated more than 15 children per month (28.7%) and termed as dentists with high volume of pediatric patients for the purpose of this study, but unfortunately it was seen that overall adherence to the AAPD guidelines was low with respect to prescribing antibiotics for treating dental infections in children. Our findings showed a lack of consistency between the manner in which dentists with low and high volume of pediatric patients treat dental infection in children which was similar to that seen in other studies [22, 24, 25].

This study shows overall adherence to professional guidelines ranging from 26.1% to 44.2% while William R. Cherry et al. in 2012 reported a similar low percentage of adherence to professional guidelines ranging from 10% to 42% [24]. K Al-Johani et al. also reported in 2017 that overall adherence to the professional guidelines ranged from 9.5% to 45% [22]. Similar finding were seen in other studies in the USA and this clearly demonstrates the increased misuse of antibiotics by dentists that may contribute to global antimicrobial resistance (AMR) [22, 25].

Yee Chen Wong et al. in 2016 reported a lack of harmony among guidelines and the antibiotic prescribing practices of dentists, in each of the clinical case scenarios according to the AAPD guidelines ranging from 15.7% to 43.5% [40]. Looking at the AAPD guidelines case scenarios in our study, in the first case only 26.8% dentists choose to prescribe antibiotics only for pain and facial swelling with radiographic evidence of pathology, which is consistent with the AAPD guidelines results seen in a study done in USA [24]. For case 2, when fever was added to the scenario, the adherence rate was still around 26.1% in our study which was much higher than seen in other studies [22, 24]. For case 3, when fever was absent, but draining fistula was added to the scenario, the adherence rate was 36.8% in our study which was slightly higher than seen in other studies done in USA and Saudi Arabia [22,24]. Scenarios 1, 2 and 3 were in-office cases and scenario 4 and 5 were weekend cases in the AAPD five case scenarios questionnaire. For case 4 and 5, the adherence rate was 44.2% and 33.9% respectively in our study which is very high to that seen in other studies [22, 24].

We hypothesized that there would be a difference in antibiotic prescribing pattern between the dentists with low and high volume of pediatric patients with later being more adherent to AAPD guidelines [22, 24], but when we compared the AAPD guideline adherence in these two groups we saw that contrary to other studies overall adherence was low in dentists with high volume of pediatric patients as compared to dentists with low volume of pediatric patients in all case scenarios in our study, probably because the later are less influenced by social factors such as family pressures of the child for quick treatment response, leading to unnecessary antibiotic use in pediatric dentists. The results of this study showed poor adherence to professional guidelines and a lack of harmony between the antibiotic prescribing patterns of dentists.

Publications have suggested that inappropriate use of antibiotics has brought about antibiotic resistance which needs to be addressed [31]. The World Health Organization (WHO) has developed strategies and provided recommendations to curb irrational use of antibiotics [32, 33]. Various studies have given proof of inappropriate practices by dentists highlighting a number of reasons, varying from insufficient updated information to social factors etc [20–25].

Locally in Pakistan, the available research on prescription patterns of antibiotics is limited at best [34–38]. One such study demonstrated the need of creating guidelines by relevant health agencies on accessible literature to modulate adequate use of antibiotics and its dissemination [39]. Whilst it is important to understand factors influencing the practitioners’ attitudes towards antibiotic prescribing, it is also necessary to provide clear guidelines based on sound clinical knowledge.

Although infectious diseases are more common in developing countries, such as Pakistan, due to poor hygienic condition of hospitals, the surgeons usually overprescribe broad spectrum antibiotics which may add to the burden of AMR [40]. A study done in Ethiopia where national antibiotic prescription guidelines were mostly not followed showed that lack of the knowledge of local resistance patterns is probably the reason for injudicious antibiotic prescription pattern seen in the developing world, [41].

Because of the absence of standard guidelines for prescribing prophylactic antibiotics in dental procedures, we used AADP guidelines to evaluate the rationality of antibiotic prescription. Programs are needed to update antibiotic prescription practices and this study highlighted the need to make such these guidelines accessible to the dentists in our country as well as across the world. Continuing dental education programs are a much needed resource for dental healthcare professionals and these will have an impact on the prescribing practices leading to reduced antibiotic resistance. Naveen N et al. in 2015 reported that out of the proportion of participants who had attended continued dental education program on antibiotics in India, 36% were adherent to professional guidelines [42]. Another indian study reported that over ninety percent of dentists admitted that over endorsing of antibiotic use will results in antimicrobial resistance and similar number routinely upgrade themselves by studying some current methodical information preceding to the utilization of antibiotics in dentistry [43].

In spite of absence of a national infection control policy, Pakistan has recently been more active on the front of tackling antimicrobial resistance [44, 45]. Comprehensible and more specific professional guidelines education is urgently needed in Pakistan that may eventually lead to improved adherence to antibiotics among health professionals including dentists. This study highlights the need to ensure that dentists are included at the time for formulating and bringing into action comprehensible guidelines to arrest further antibiotic abuse by both medical practitioners and dentists. There is an immediate need for the proper dissemination of information regarding prescribing guidelines as well as instructive strategies to encourage the proper and suitable utilization of medications in dentistry.

## Conclusion

This study highlights the fact that there is an urgent need to generate professional awareness regarding the risks of injudicious use of antibiotics among dentists as majority of our participant dentists did not follow the AAPD guidelines for prescription of antibiotics. It is recommended that appropriate professional guidelines for antibiotic use and misuse should be specified to counter drug resistance along with regular continuing dental education programs to update and revise the pattern of antibiotic prescription.

## Supporting information

S1 Appendix (DOCX)
