# Supplementary material for: Antibiotic prescription patterns for treating dental infections in children among general and pediatric dentists in teaching institutions of Karachi, Pakistan
Source: PLoS One. 2020 Jul 10;15(7):e0235671. doi: 10.1371/journal.pone.0235671 (PMC7351155; doi:10.1371/journal.pone.0235671)
Supplement: S1 Appendix — (DOCX) [file pone.0235671.s001.docx]

## APPENDIX : QUESTIONNAIRE

**Antibiotics prescription pattern for treating dental infections in children among dental practitioners and post graduates in teaching institution of Karachi, Pakistan.**

**DEMOGRAPHICS AND PRACTICE CHARACTERISTICS OF PARTICIPATING DENTISTS**

Date:

Study ID: ___ ______

- **Gender** Male____ Female_____
- **Age** ______ Years
- **Educational qualification** Dentist____ PGs (MDS, MCPS, FCPS, PHD, OTHER)
- **Experience after graduation** ______ Years, ______ Months
- **Practice location** District____ Township______
- **Practice type**

Private practice / Public practice_____ Academic institution _____

Hospital dentistry_____ Primary Health centers_____ Any Other____________

- **Type of Antibiotic prescribed**

Amoxicillin____ Penicillin____

Clindamycin____ Cephalexin____ Any other_____________

- **How many children do you treat per month?**

Less than 15____ More than 15____

- **How often do you write prescriptions for antibiotics for dental infections?**

Daily____ Weekly____

Monthly____ Hardly ever____

**Case scenarios (Please tick only one option for each case)**

**Case 1**

A healthy (ASA I) 9-year-old child, who is a patient of record, visits your office during regular business hours with tooth pain in the lower right quadrant. On clinical examination, you notice a deep carious lesion on tooth number 85 (mandibular right primary second molar). Would you prescribe antibiotics for the following:
[ ] Pain only?

[ ] Symptoms of pain + local swelling + no radiographic evidence of pathology?

[ ] Symptoms of pain + local swelling + radiographic evidence of pathology?

[ ] Symptoms of pain + facial swelling + radiographic evidence of pathology?

**Case 2**
A healthy (ASA I) 9-year-old child, who is a patient of record, visits your office during regular business hours with tooth pain in the lower right quadrant and a fever of 101 F. On clinical examination, you notice a deep carious lesion on tooth number 85 (mandibular right primary second molar). Would you prescribe antibiotics for the following:
[ ] Pain and fever?

[ ] Symptoms of pain + local swelling + no radiographic evidence of pathology?

[ ] Symptoms of pain + local swelling + radiographic evidence of pathology?

[ ] Symptoms of pain + facial swelling + radiographic evidence of pathology?

**Case 3**
A healthy (ASA I) 9-year-old child, who is a patient of record, visits your office during regular business hours with tooth pain in the lower right quadrant. The child has no fever. On clinical examination, you notice a deep carious lesion on tooth number 85 (mandibular right primary second molar) along with a draining fistula. Would you prescribe antibiotics for the following:
[ ] Pain only?

[ ] Symptoms of pain + local swelling + no radiographic evidence of pathology?

[ ] Symptoms of pain + local swelling + radiographic evidence of pathology?

[ ] Symptoms of pain + facial swelling + radiographic evidence of pathology?

**Case 4**
The parent of a healthy (ASA I) 9-year-old child, who is a patient of record, calls you on a Saturday afternoon because the child has a chief complaint of tooth pain in the lower right quadrant. Would you prescribe antibiotics for the following:
[ ] Pain only?

[ ] Symptoms of pain + local swelling?

[ ] Symptoms of pain + facial swelling?

[ ] Would you see the child before prescribing antibiotics?

**Case 5**
The parent of a healthy (ASA I) 9-year-old child, who is a patient of record, calls you on a Saturday afternoon and reports that the child has pain on the lower right quadrant with some warmness of the skin and some swelling that she noticed that morning. Would you prescribe antibiotics for the following:
[ ] Pain only?
[ ] Symptoms of pain + warmness of the skin?
[ ] Symptoms of pain + warmness of the skin + localized swelling?
[ ] Symptoms of pain + warmness of the skin + facial swelling?
[ ] Would you see the child before prescribing antibiotics?
